# Accuracy of markerless motion capture in assessing sagittal-plane lower limb kinematics during running: a systematic review and meta-analysis

**DOI:** 10.3389/fbioe.2026.1915559

**Published:** 2026-10-12

**Authors:** Ming Gao, Yilin An, Conghua Feng, Zhiyuan Yang, Zhenyang Xu, Dewei Mao, Qinghui Shang, Zhiyuan Sun

**Affiliations:** 1 Shandong Sport University, Jinan, Shandong, China; 2 Beijing Sport University, Beijing, China; 3 Tianjin University of Sport, Tianjin, China; 4 Division of Physical Education, The Chinese University of Hong Kong, Shenzhen, China

**Keywords:** kinematics, markerless motion capture, meta-analysis, running, sagittal plane, measurement error, lower limb

## Abstract

**Background:**

Markerless motion capture (MMC) systems have gained increasing attention in sports science and clinical assessment. However, their accuracy in measuring sagittal-plane lower limb kinematics during running remains unclear.

**Methods:**

A systematic review and meta-analysis was conducted following established guidelines. MEDLINE, Embase, Cochrane Library, Web of Science, and Scopus were searched for studies comparing MMC with marker-based systems (MBSs). Data were analyzed using a random-effects model in R 4.3.3. Risk of bias was assessed using the COSMIN Risk of Bias checklist for measurement error and the Joanna Briggs Institute (JBI) Critical Appraisal Checklist for Analytical Cross-Sectional Studies. Heterogeneity, sensitivity analyses, and publication bias (Egger’s and Begg’s tests) were also evaluated. Subgroup analyses were conducted to explore potential sources of technical heterogeneity according to sampling frame rate, number of cameras, and running condition.

**Results:**

A total of 8 studies were included in this analysis. The pooled root mean square error (RMSE) was 3.84° (95% CI: 2.53–5.15°) for the hip, 4.30° (95% CI: 2.55–6.05°) for the knee, and 3.93° (95% CI: 2.29–5.58°) for the ankle. These findings indicated relatively small measurement errors for sagittal-plane lower limb kinematics, although the magnitude and uncertainty of errors varied across joints. Sensitivity analyses generally supported the robustness of the findings. For knee joint kinematics, funnel plot asymmetry tests did not provide statistically significant evidence of publication bias; however, given the limited number of studies, these findings should be interpreted cautiously. Subgroup analyses indicated that technical characteristics had joint-specific effects on MMC-derived kinematic errors, with no consistent advantage observed for higher frame rates or greater camera numbers.

**Conclusion:**

The included MMC systems showed relatively small measurement errors for sagittal-plane lower limb kinematics during running. These findings are specific to the MMC technologies and running conditions evaluated in this review.

## 1 Introduction

The benefits of running in promoting chronic disease prevention and overall physical and mental health have been widely recognized. From a biomechanical perspective, analyzing lower limb joint kinematics and load distribution during running has substantial scientific and clinical significance for elucidating patterns of neuromuscular coordination and assessing the risk of sports-related injuries (Subotnick, 1985; Ceyssens et al., 2019). Running is characterized by cyclical forward body motion, with the sagittal plane serving as the key dimension for assessing lower limb joint range of motion and detecting gait abnormalities (Grant AD. Gait Analysis, 2010). Recently, the functional characteristics of running have been increasingly applied in clinical screening, including the assessment of lower limb musculoskeletal disorders, neuromuscular dysfunction, and rehabilitation outcomes, thereby providing reliable quantitative metrics for individualized interventions (Peterson et al., 2022; Munteanu and Barton, 2011).

In the field of kinematic measurement, marker-based motion capture systems (MBSs), which rely on reflective markers placed on bony landmarks, have long been widely used as a reference method for obtaining high-precision three-dimensional spatial trajectory data under controlled laboratory conditions (Kanko et al., 2021a; Moro et al., 2022). However, their practical application is hindered by high demands on spatial configuration and optical conditions. More critically, relative movement between markers and underlying bones generates soft tissue artifacts (STA), a primary source of systematic error in skeletal motion measurements (Wade et al., 2022; Peters et al., 2010). This phenomenon is particularly pronounced in high-dynamic tasks such as running, and current biomechanical algorithms remain insufficient to fully compensate for or eliminate it.

In response, markerless motion capture (MMC) has emerged as a cutting-edge measurement approach that has garnered considerable academic attention. This technology does not require physical markers on the participant’s body; instead, it relies on multi-view video streams combined with deep learning algorithms to identify skeletal key points and anatomical structures. Leveraging its significant non-invasive advantage, MMC has increasingly penetrated the field of sports science, offering a novel technical avenue for kinematic analysis under high-dynamic and naturalistic movement conditions (Lam et al., 2023). Existing studies indicate that MMC demonstrates good agreement in gait analysis and acceptable measurement accuracy in jumping tasks. However, its performance in assessing sagittal-plane lower limb kinematics during the higher-dynamic task of running requires further validation, which has limited its broader application in clinical screening and rehabilitation practice (Scataglini et al., 2024; Ogura et al., 2025). To date, most studies have focused on validating MMC in gait or jumping tasks, and a systematic meta-analysis of its accuracy in measuring sagittal-plane lower limb kinematics during running is still lacking. This study conducted a meta-analysis to integrate and compare MMC measurements with those of a reference motion capture system in running tasks, thereby providing evidence to inform the wider adoption and application of MMC.

## 2 Methods

The study protocol was initially registered on the Open Science Framework (OSF) on 8 March 2026 (Version 1), and updated on 31 August 2026 (Version 2). The update clarified the eligibility criteria to explicitly include only three-dimensional markerless motion capture systems, resulting in the exclusion of two-dimensional systems. The primary research question and objectives remained unchanged. The full protocol and version history are available at https://doi.org/10.17605/OSF.IO/785EP (Foster and Deardorff, 2017).

### 2.1 Search strategy

To comprehensively identify studies meeting the inclusion criteria, a systematic search was conducted in MEDLINE, Embase, Cochrane Library, Web of Science, and Scopus, covering the period up to March 2026. The search strategy encompassed core topics including “markerless motion capture,” “marker-based motion capture,” “3D motion analysis,” “accuracy,” “reliability,” and “measurement error.” In addition to the database searches, supplementary manual search and citation tracking of the reference lists of the included studies were performed to minimize the risk of missing relevant literature, thereby ensuring comprehensive coverage of eligible sources. The literature search and screening process was conducted in accordance with the PRISMA 2020 guidelines. The completed PRISMA 2020 checklist is provided in Supplementary Material, while the detailed search strategies are provided in Supplementary Table S1. Reasons for exclusion at the full-text screening stage are provided in Supplementary Table S2.

### 2.2 Inclusion and exclusion criteria

Inclusion Criteria: Studies were included if they met all of the following criteria (Subotnick, 1985): participants were healthy adults able to perform running tasks independently (Ceyssens et al., 2019); the study employed 3D MMC system and MBS, with direct comparison of their three-dimensional measurements (Grant AD. Gait Analysis, 2010); the article was published in English (Peterson et al., 2022); the study reported clear quantitative error metrics and was an original, peer-reviewed research article. No minimum sample size was prespecified because small-sample validation studies may provide relevant information on measurement accuracy.

Exclusion Criteria (Subotnick, 1985): animal experiments or computational simulations (Ceyssens et al., 2019); focused on populations with specific diseases or interventional treatments or did not represent typical running gait (Grant AD. Gait Analysis, 2010); reviews, guidelines, or other non-original research (Peterson et al., 2022); lacked key data, preventing extraction of complete effect sizes (Munteanu and Barton, 2011); studies using 2D MMC systems.

### 2.3 Data extraction and quality assessment

Data extraction and methodological quality assessment were independently conducted by two researchers (GM and YZY) to ensure completeness, accuracy, and consistency of the information. The extracted data included basic study information, participant characteristics (sample size, age, sex), descriptions of the running tasks, types of motion capture systems used, and the measurement metrics along with their corresponding statistical data. The RMSE/RMSD values reported by the original studies for sagittal-plane lower limb kinematics during running were directly extracted and used as the effect measures in the meta-analysis. The risk of bias in the assessment of measurement error was evaluated using the COSMIN Risk of Bias checklist (Mokkink et al., 2018). Although COSMIN has been widely applied to patient-reported outcomes, its framework also provides criteria for assessing measurement properties, including measurement error. As the primary outcome of this meta-analysis was RMSE/RMSD, reflecting measurement error, only the COSMIN items relevant to measurement error were assessed. Studies were rated as Very Good, Adequate, Doubtful, or Inadequate. In addition, the methodological risk of bias of the included cross-sectional studies was independently assessed using the Joanna Briggs Institute (JBI) Critical Appraisal Checklist for Analytical Cross-Sectional Studies (Barker et al., 2026). This assessment was performed to evaluate the methodological quality of the individual studies beyond the measurement-error-specific assessment provided by COSMIN. Any disagreements in the quality assessment were resolved through discussion with a third researcher (SQH).

### 2.4 Statistical analysis

All data from the included studies were analyzed using R 4.3.3 with the metafor package. The primary effect measure in this meta-analysis was the root mean square error (RMSE) for each joint. For random-effects meta-analyses, 95% prediction intervals (PIs) were calculated to account for between-study heterogeneity and to estimate the range within which the true effect of a future comparable study may lie. Root mean square deviation (RMSD) uses the same root-mean-square formulation as RMSE when calculated from the deviations between the MMC and reference-system measurements; therefore, RMSE and RMSD were treated as equivalent measures of measurement error in this analysis. The RMSE/RMSD values reported by the original studies were directly extracted and used as the effect measures. RMSE/RMSD were calculated from point-by-point differences between the MMC and reference-system measurements across the corresponding kinematic time-series data. Methodological details regarding joint angle definitions, coordinate systems, filtering procedures, and gait-cycle normalization were not consistently reported across the included studies. Because none of the included studies reported the standard deviation, standard error, or 95% confidence interval of RMSE/RMSD, an approximate standard error was calculated from the reported RMSE/RMSD and sample size as follows:

$$\left[SE_{i}\approx \frac{RMSE_{i}}{\sqrt{n_{i}}}\right]$$

where $\left(SE_{i}\right)$ is the estimated standard error, $\left(RMSE_{i}\right)$ is the RMSE/RMSD reported by study $\left(i\right)$ , and $\left(n_{i}\right)$ is the corresponding sample size. The sampling variance was calculated as:

$$\left[V_{i}=SE_{i}^{2}\right]$$

Given the heterogeneity in methodology and participant characteristics among the included studies, a random-effects model was employed to pool RMSE/RMSD values for each joint. Between-study variance $\left(\left(\tau^{2}\right)\right)$ was estimated using restricted maximum likelihood (REML). The random-effects weight for each study was calculated as:

$$\left[w_{i}=\frac{1}{V_{i}+\tau^{2}}\right]$$

The pooled RMSE/RMSD was calculated as:

$$\left[\hat{\mu }=\frac{\sum_{i=1}^{k}w_{i}RMSE_{i}}{\sum_{i=1}^{k}w_{i}}\right]$$

with its standard error calculated as:

$$\left[SE\left(\hat{\mu }\right)=\sqrt{\frac{1}{\sum_{i=1}^{k}w_{i}}}\right]$$

and the 95% confidence interval calculated as:

$$\left[\hat{\mu }\pm 1.96\times SE\left(\hat{\mu }\right)\right]$$

The above standard error estimation was an approximate approach necessitated by the absence of directly reported uncertainty measures in the included studies. Heterogeneity was quantitatively assessed using the I^2^ statistic, with I^2^ < 25% indicating low heterogeneity, 25%–50% moderate heterogeneity, 50%–75% substantial heterogeneity, and 75%–100% considerable heterogeneity (Huedo-Medina et al., 2006). To explore potential sources of technical heterogeneity, subgroup analyses were conducted according to three MMC characteristics: sampling frame rate (≤60 Hz vs. >60 Hz) (Mundt et al., 2024), number of cameras (≤8 vs. >8 cameras) (Yang and Jung, 2025), and running condition (treadmill vs. overground running) (Van Hooren et al., 2023). These factors were selected based on their availability and potential influence on motion capture accuracy. Subgroup differences were assessed to determine whether measurement accuracy varied across these technical conditions.

To evaluate the potential influence of individual studies on the overall results, sensitivity analyses were performed by excluding influential studies and recalculating the pooled effect sizes for each joint, thereby assessing the robustness of the findings. Additionally, to explore potential publication bias, funnel plots were constructed and supplemented with statistical assessment using Egger’s test and Begg’s test (Egger et al., 1997; Begg and Mazumdar, 1994). All analyses were performed separately for the hip, knee, and ankle joints to allow independent evaluation of RMSE for each joint. The results of the meta-analysis were visualized using forest plots, with RMSE presented in 5° increments, using 5° as a commonly accepted reference threshold to facilitate the interpretation and comparison of measurement errors across studies (McGinley et al., 2009).

## 3 Results

### 3.1 Study characteristics

The initial search identified a total of 5,429 records. After removing 3,397 duplicates, 2,032 records were screened by title and abstract. Following preliminary screening, 1,955 records were excluded for not meeting the inclusion criteria, leaving 77 articles for full-text assessment. Ultimately, based on the predefined inclusion and exclusion criteria, 8 studies were included in the meta-analysis (Needham et al., 2022; Pagnon et al., 2022; Song et al., 2023; Verheul et al., 2026; Kanko et al., 2024; Ceriola et al., 2024; Remy et al., 2025; Needham, 2026) (Figure 1). Detailed characteristics and full list of the included studies are presented in Table 1. Specifically, all 8 studies reported sagittal-plane RMSE for the hip, 7 for the knee, and 6 for the ankle. Among the studies reporting corresponding 95% CIs, three studies for the hip, one for the knee, and one for the ankle had RMSE estimates with 95% CIs entirely below the 5° reference threshold.

**FIGURE 1:**
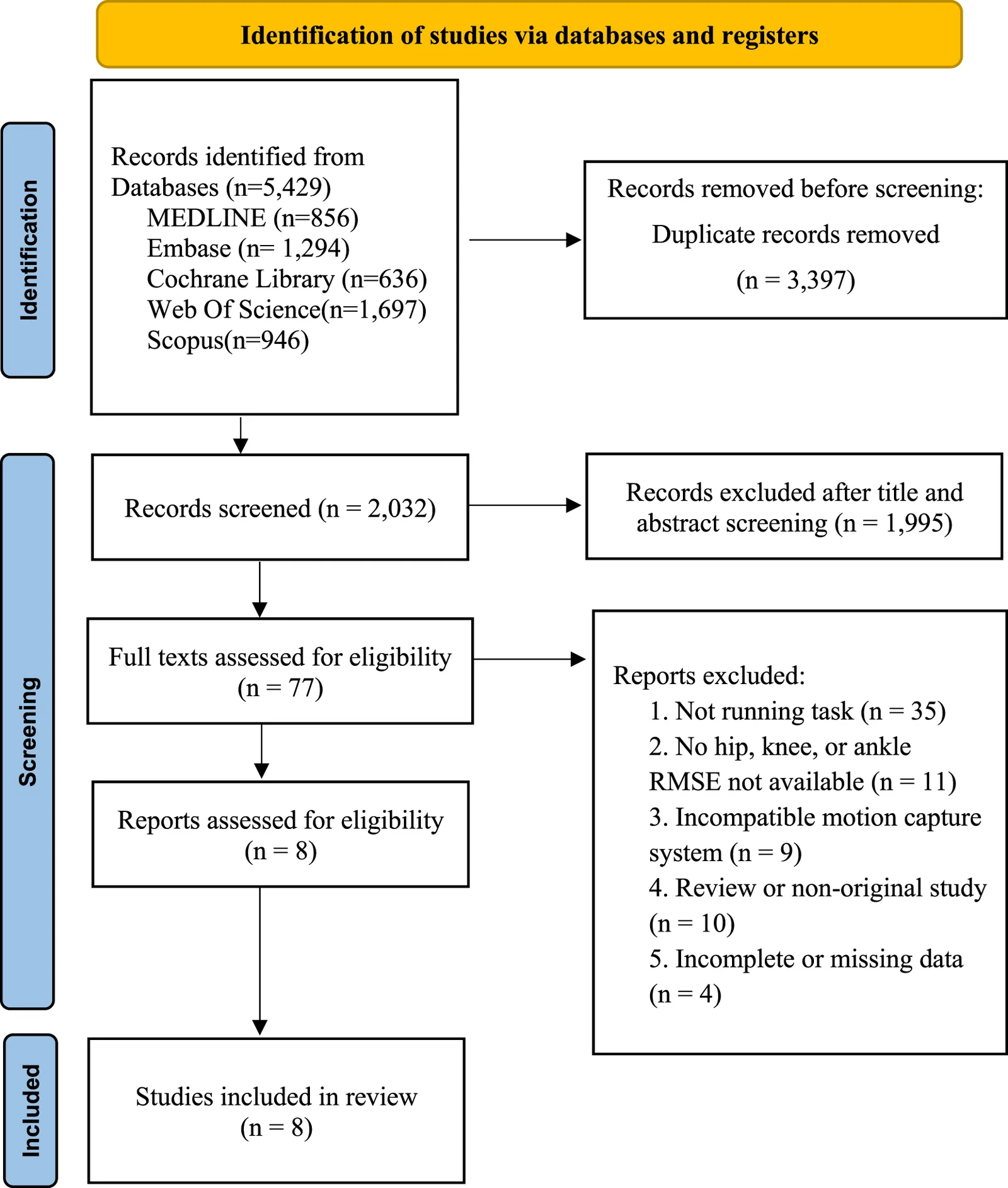
PRISMA flow diagram of literature screening and study inclusion.

**TABLE 1:** The basic characteristics of the included studies.

Author	Country	Population	Numbers	Motion capture system (markerless)	Reference system (marker-based)	Joint analyzed	Accuracy metric
Needham et al., 2022	United Kingdom	Healthy adults	15 7M/8F	9 JAI sp5000c cameras (1920x1080, 200 Hz), using the OpenPose algorithm	15-Camera qualisys oqus system, 44 markers +4 clusters, 200 Hz	Hip	RMSE
Pagnon et al., 2022	France	Healthy adults	1 1M/0F	68 video cameras +8 virtual cameras (30 Hz), using OpenPose (BODY_25B model)	83 markers (35 anatomical +12 clusters), qualisys system; 30 Hz	Hip Knee Ankle	RMSE
Song et al., 2023	United States	Healthy adults	10 5M/5F	8 high-resolution video cameras (optitrack prime color), using Theia3D software, 100 Hz	12 near-infrared cameras (Raptor-E x10, Raptor-12 x2), 31 retro-reflective markers, 100 Hz	Hip Knee Ankle	RMSD
Verheul et al., 2026	United Kingdom	Healthy adults	30 19M/11F	OpenCap system (3 iPads), using HRNet + marker-augmented model with OpenSim IK; 240 Hz	Vicon vantage system, 71 markers, processed with OpenSim inverse kinematics (IK); 200 Hz	Hip Knee Ankle	RMSD
Kanko et al., 2024	Canada	Healthy adults	30 15M/15F	8 qualisys miqus cameras, analyzed with Theia3D for 3-DOF hip/knee/ankle kinematics; 85 Hz	7 qualisys oqus cameras, 71 markers, with Visual3D IK; 85 Hz	Hip Knee Ankle	RMSD
Ceriola et al., 2024	Italy	Healthy adults	10 10M/0F	2 logitech BRIO 4K webcams, synchronized via OBS studio, using a DNN-based pose estimator; 60 Hz	6 vicon Vero2.2 infrared cameras, 39 reflective markers, using the plug-in gait full-body model; 120 Hz	Hip Knee	RMSD
Remy et al., 2025	France	Healthy adults	3 2M/1F	9 qualisys miqus cameras, processed using the Pose2Sim workflow (RTMLib, HALPE_26 model) with OpenSim IK; 60 Hz	23 qualisys oqus cameras, processed using the rajagopal musculoskeletal model and OpenSim IK; 120 Hz	Hip Knee Ankle	RMSE
Needham et al., 2021	United Kingdom	Healthy adults	6 3M/3F	9 JAI sp5000c cameras, OpenPose + multi-view 3D fusion, kalman smoothing, OpenSim IK; 200HZ	15-Camera qualisys oqus system, 44 markers +4 clusters, using a 6DOF full-body model; 200 Hz	Hip Knee Ankle	RMSE

### 3.2 Quality assessment

This study systematically evaluated the “Measurement Error” dimension of the included studies using the COSMIN Risk of Bias checklist, with itemized scoring applied separately for the hip, knee, and ankle joint measurements (Table 2). The assessment indicated that the overall methodological quality of the included studies was good: 17 evaluation items were rated as “Adequate”, while 4 items were rated as “Doubtful” due to higher variability for specific joints, primarily reflecting insufficient reporting of measurement variability in the respective studies. Second, the methodological risk of bias of the individual included cross-sectional studies was assessed using the JBI Critical Appraisal Checklist for Analytical Cross-Sectional Studies. Overall, six studies were rated as having a high risk of bias and two studies as having a moderate risk of bias (Supplementary Table S3).

**TABLE 2:** COSMIN risk of bias assessment for measurement error of the included studies.

Study	Measurement error		
	Hip	Knee	Ankle
Needham et al., 2022	A	-	-
Lambricht et al., 2025	A	A	A
Song et al., 2023	A	D	A
Verheul et al., 2026	A	A	A
Kanko et al., 2024	A	A	A
Ceriola et al., 2024	A	A	-
Remy et al., 2025	D	D	D
Needham et al., 2021	A	A	A

A, adequate; D, doubtful; –, no relevant data available.

### 3.3 Meta-analysis

#### 3.3.1 Hip joint RMSE

A total of 8 studies reported sagittal-plane RMSE data for the hip joint. The random-effects meta-analysis showed a pooled RMSE of 3.84° (95% CI: 2.53–5.15°) for the hip, with a 95% prediction interval of 0.80–7.07, indicating moderate heterogeneity (I^2^ = 59.50%). The corresponding forest plot is presented in Figure 2A. Among the included studies, six reported RMSE values below the commonly used reference threshold of 5°, with three studies having 95% confidence intervals entirely below this threshold.

**FIGURE 2:**
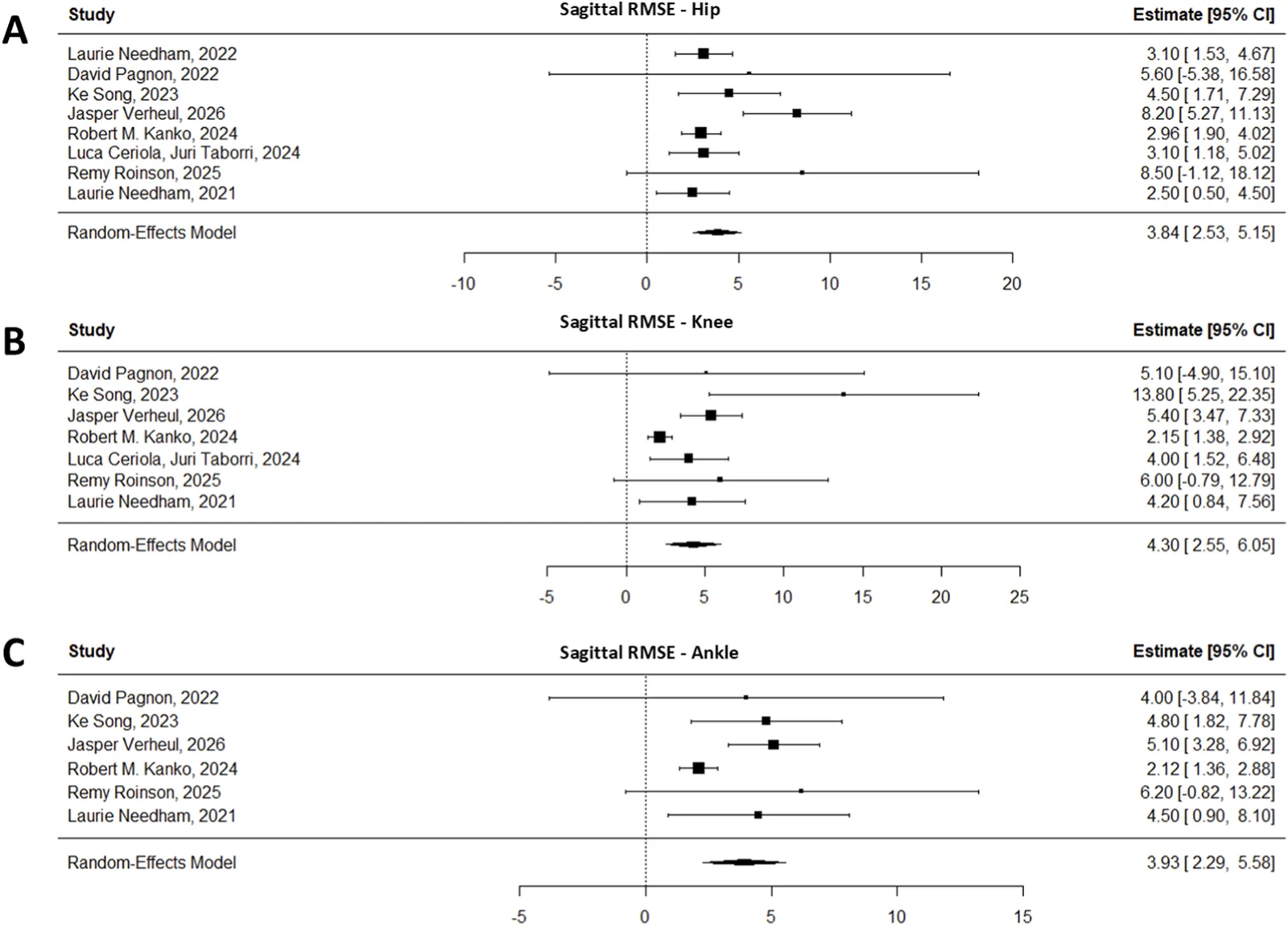
Random-effects model forest plots of sagittal-plane root mean square error (RMSE) for the hip, knee, and ankle joints. Note: **(A)** Sagittal RMSE – Hip; **(B)** Sagittal RMSE – Knee; **(C)** Sagittal RMSE – Ankle. RMSE, root mean square error.

#### 3.3.2 Knee joint RMSE

A total of 7 studies reported sagittal-plane RMSE data for the knee joint. The random-effects meta-analysis showed a pooled RMSE of 4.30° (95% CI: 2.55–6.05°) for the knee, with a 95% prediction interval of 0.62–8.51°, indicating moderate heterogeneity (I^2^ = 59.77%), as shown in the forest plot (Figure 2B). Among the included studies, only two reported RMSE values below the commonly used error threshold of 5°.

#### 3.3.3 Ankle joint RMSE

A total of 6 studies reported sagittal-plane RMSE data for the ankle joint. The random-effects meta-analysis showed a pooled RMSE of 3.93° (95% CI: 2.29–5.58°) for the ankle, with a 95% prediction interval of 1.29–6.90°, indicating moderate heterogeneity (I^2^ = 55.73%), as illustrated in the forest plot (Figure 2C). Among the included studies, five reported RMSE values below the commonly used error threshold of 5°.

### 3.4 Publication bias

To assess potential publication bias, Egger’s regression test and Begg’s rank correlation test were conducted for the included studies, complemented by visual inspection of funnel plots. For the hip joint, no significant publication bias was detected (Egger’s test: p = 0.4936; Begg’s test: p = 0.2366). For the knee joint, Egger’s test indicated a potential risk of publication bias (p = 0.0211), suggesting that the results should be interpreted with caution; however, Begg’s test did not show significant bias (p = 0.2397). For the ankle joint, neither Egger’s test (p = 0.0953) nor Begg’s test (p = 0.7461) indicated significant funnel plot asymmetry. Given the limited number of studies, these tests were considered exploratory, and the results should therefore be interpreted cautiously (Figure 3).

**FIGURE 3:**
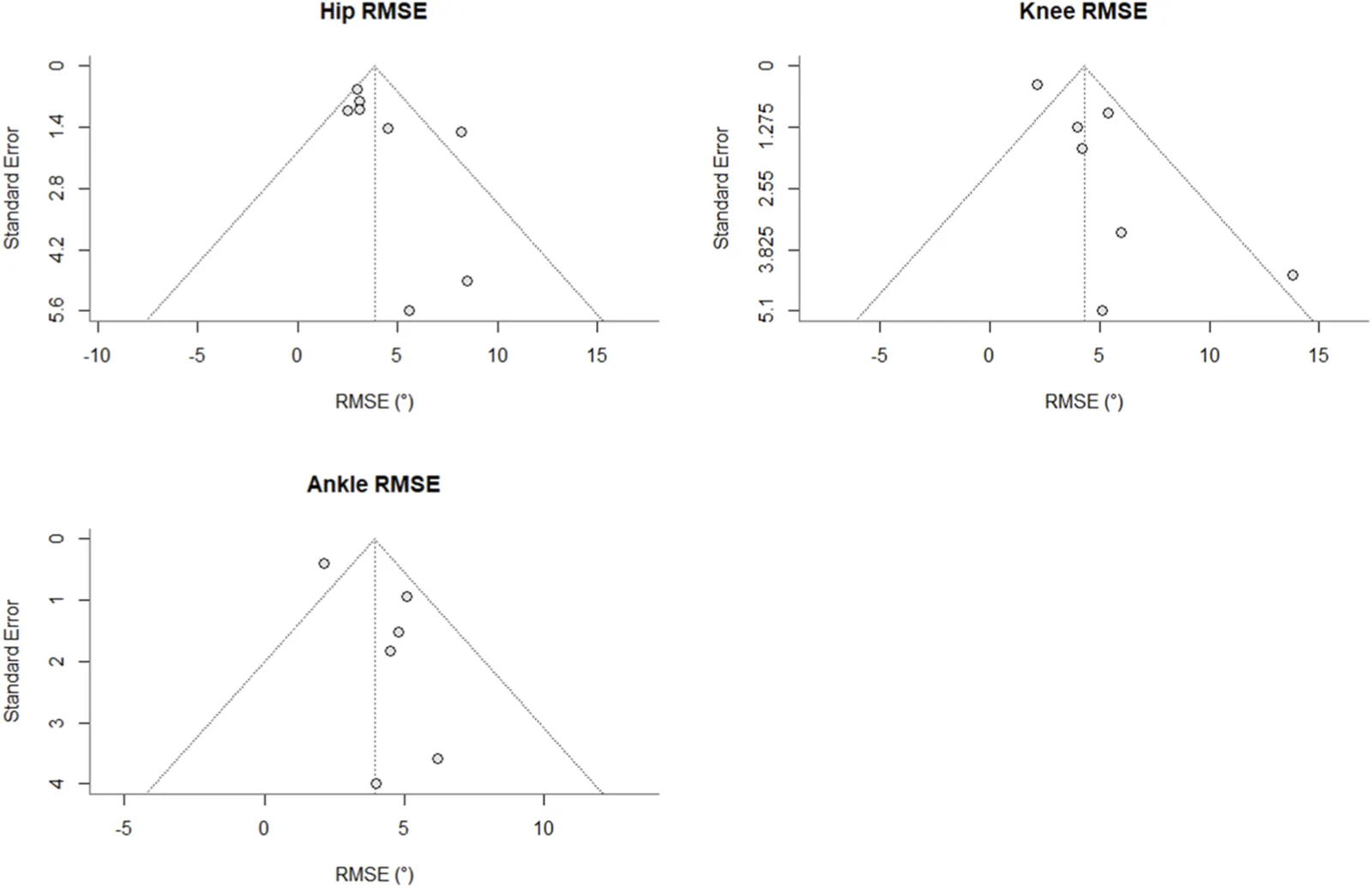
Funnel plots of sagittal-plane kinematic RMSE for the hip, knee, and ankle joints.

### 3.5 Subgroup analysis

#### 3.5.1 Sampling frame rate

Subgroup analysis by sampling frame rate showed that the pooled RMSE was 3.37° (95% CI: 1.52–5.23) for the hip, 4.28° (95% CI: 2.01–6.55) for the knee, and 5.57° (95% CI: 1.38–9.76) for the ankle in the ≤60 Hz group (Supplementary Figure S1). In the >60 Hz group, the pooled RMSE was 3.97° (95% CI: 2.21–5.72) for the hip, 4.65° (95% CI: 1.83–7.47) for the knee, and 3.83° (95% CI: 2.06–5.60) for the ankle (Supplementary Figure S2). These findings indicated comparable measurement errors across the two frame-rate categories, although the pooled estimates varied across joints.

#### 3.5.2 Number of cameras

Subgroup analysis by the number of cameras showed that, in the ≤8-camera group, the pooled RMSE was 4.43° (95% CI: 2.21–6.65) for the hip, 4.49° (95% CI: 1.89–7.10) for the knee, and 3.76° (95% CI: 1.65–5.87) for the ankle (Supplementary Figure S3). In the >8-camera group, the pooled RMSE was 3.00° (95% CI: 1.78–4.21) for the hip, 4.60° (95% CI: 1.72–7.48) for the knee, and 4.73° (95% CI: 1.77–7.70) for the ankle (Supplementary Figure S4). Overall, the pooled measurement errors varied across joints according to the number of cameras, with lower hip RMSE but higher ankle RMSE in the >8-camera group, while knee RMSE was comparable between the two groups.

#### 3.5.3 Running condition

Subgroup analysis by running condition showed that, during overground running, the pooled RMSE was 3.24° (95% CI: 2.12–4.35) for the hip, 6.22° (95% CI: 2.45–9.99) for the knee, and 4.77° (95% CI: 2.67–6.87) for the ankle (Supplementary Figure S5). During treadmill running, the pooled RMSE was 4.53° (95% CI: 1.38–7.67) for the hip, 3.67° (95% CI: 1.64–5.71) for the knee, and 3.49° (95% CI: 0.58–6.40) for the ankle (Supplementary Figure S6). Overall, measurement errors varied across joints according to running condition, with lower RMSE during overground running for the hip but lower RMSE during treadmill running for the knee and ankle.

### 3.5.4 Sensitivity analysis

To evaluate the influence of individual studies on the pooled effect sizes, sensitivity analyses were conducted separately for the hip, knee, and ankle joints. After excluding influential studies, the pooled RMSE for the hip slightly decreased from 3.84° to 3.73°, showing minimal change; for the knee, the pooled RMSE slightly decreased to 3.72° after removing the corresponding studies; similarly, for the ankle, exclusion of extreme studies resulted in a pooled RMSE of 3.83° (Supplementary Figure S7). Overall, the changes in pooled RMSE estimates were small across all three joints, suggesting limited influence of individual studies on the overall measurement error estimates.

## 4 Discussion

This systematic review and meta-analysis quantified measurement error of MMC for sagittal-plane kinematics of key lower-limb joints during running. The results indicated that MMC showed relatively low measurement errors overall for the hip, knee, and ankle joints, with pooled RMSE values generally indicating relatively low measurement errors. However, the substantial variation in sample size, particularly the inclusion of very small samples, may reduce the precision and stability of the pooled estimates and contribute to greater uncertainty in the findings. These findings suggest that MMC may have potential for assessing sagittal-plane lower-limb kinematics during running and may provide a practical option for motion assessment in sports science and clinical gait research.

The results indicate that, although MMC showed relatively low overall measurement error during running, variability across studies persists, suggesting that MMC measurements may still differ from those obtained using marker-based reference systems under different measurement conditions. Previous studies have also reported systematic discrepancies between MMC and marker-based systems across different joints, kinematic variables, and running conditions, suggesting that measurement error may vary depending on the measurement context (Verheul et al., 2026; Kanko et al., 2024; Tang et al., 2024). A descriptive examination of the included studies also suggested that measurement error may vary across MMC systems; however, the limited number of studies and substantial differences in system configurations precluded a robust comparison between system types. When fewer than 8 studies were included, Egger’s and Begg’s tests had limited statistical power and were therefore considered exploratory; nonsignificant results should not be interpreted as evidence of an absence of publication bias.

The measurement performance of motion capture systems may be influenced by hardware configuration and data acquisition protocols, including camera geometry, image resolution, temporal sampling frequency, and system calibration procedures (Yoma et al., 2025; Unsihuay and Clavo, 2026; Lee et al., 2025). Differences in these technical parameters may contribute to between-study heterogeneity and variation in kinematic measurements under dynamic movement conditions.

Second, when capturing high-dynamic motion, the spatiotemporal consistency of keypoint detection in skeletal reconstruction algorithms may exhibit fluctuations (Wade et al., 2022; Needham et al., 2021). This reflects the current limitations of existing algorithms in adapting to anatomical constraints when handling high-velocity movements and rapid joint rotations, potentially leading to artifacts or coordinate drift in kinematic outputs.

Additionally, during running, the high frequency of limb oscillations generates rhythmic displacements that place greater demands on real-time tracking algorithms. Rapid changes in limb velocity, particularly during the late swing phase, may increase keypoint localization errors (de Borba et al., 2025; Fukuchi et al., 2019). Moreover, individual morphological variations, self-occlusion, clothing interference, and surface texture can all hinder the robust identification of anatomical landmarks by the algorithm (Fiorentino et al., 2017; Ancillao et al., 2021; Ancillao et al., 2022; Metcalf et al., 2020). Although MMC avoids errors caused by marker displacement, soft tissue vibration and deformation under high-dynamic conditions can still interfere with the localization of virtual joint centers. Nevertheless, given its operational convenience and lower cost, MMC may offer potential for applications in sports monitoring and rehabilitation assessment, although further validation is needed.

To further explore potential sources of technical heterogeneity, subgroup analyses were performed according to sampling frame rate (≤60 Hz vs. >60 Hz), number of cameras (≤8 vs. >8 cameras), and running condition (treadmill vs. overground running). The findings indicated that these technical characteristics may contribute to variations in MMC-derived kinematic errors, although their effects were not consistent across joints. Specifically, differences in sampling frame rate and camera number did not show a consistent relationship with measurement error, suggesting that hardware specifications alone may not fully explain the variability in MMC performance (Walker et al., 2026). In addition, the influence of running condition varied across joints, indicating that experimental settings may contribute to differences in kinematic estimation errors (Verheul et al., 2026). Overall, MMC performance is likely determined by the interaction of multiple factors, including camera configuration, pose estimation algorithms, calibration procedures, and biomechanical modeling. Future studies should adopt standardized reporting and validation protocols to facilitate comparisons between different MMC systems.

Compared with non-periodic tasks involving jumps or directional changes accompanied by intense, multi-planar movements, running exhibits more regular temporal patterns and is predominantly sagittal-plane dominant. These characteristics of running may reduce the challenges associated with keypoint tracking compared with more complex, non-periodic movements, potentially contributing to the observed measurement performance of MMC in sagittal-plane running tasks (Novacheck, , 1998; Lachniet et al., 2018). Such relatively consistent spatiotemporal characteristics could potentially facilitate keypoint localization and reduce fluctuations in joint-angle estimation (Wilson and Likens, 2023; Mann and Hagy, 1980; van Oeveren et al., 2024). In contrast to MBSs, which are labor-intensive, highly intrusive to natural movement, and largely confined to laboratory settings, MMC requires no marker attachment. This not only greatly shortens experimental preparation time but also, owing to its compatibility with everyday clothing and lower cost, these characteristics suggest potential applications in sports monitoring, rehabilitation assessment, and movement assessment outside laboratory settings, although further validation under real-world conditions is required (Wade et al., 2022; Nishikawa et al., 2025; Espitia-Mora et al., 2024; Kanko et al., 2021b).

### 4.1 Limitations

This systematic review and meta-analysis evaluated the measurement error of MMC relative to MBSs for sagittal-plane lower-limb kinematics during running. However, several limitations should be acknowledged (Subotnick, 1985). The relatively small number of included studies, substantial variation in sample size including very small samples, and heterogeneity in experimental design, system configuration, and algorithm models may reduce the precision and stability of the pooled RMSE estimates and increase uncertainty in the findings (Ceyssens et al., 2019). The MMC systems and pose estimation algorithms varied across studies; differences in keypoint detection and skeletal reconstruction performance could influence result comparability (Grant AD. Gait Analysis, 2010). Methodological details, including joint angle definitions, coordinate systems, filtering procedures, and gait-cycle normalization, varied across studies and were therefore interpreted according to the methods reported in each original study without imposing uniform assumptions (Peterson et al., 2022). This analysis focused exclusively on sagittal-plane kinematic parameters, leaving the measurement accuracy in other movement planes unassessed (Munteanu and Barton, 2011). Although funnel plots appeared generally symmetrical, the interpretation of Egger’s and Begg’s tests should be cautious when fewer than 10 studies were included because of their limited statistical power. In addition, several analyses showed high I^2^ values, indicating that the findings should be interpreted with caution.

## 5 Conclusion

This study systematically evaluated the measurement error of MMC relative to marker-based reference systems for sagittal-plane lower-limb kinematics during running. The pooled results indicate that MMC demonstrated relatively low measurement errors for the hip, knee, and ankle, although the magnitude of error varied across joints and studies. These findings support the potential use of MMC for sagittal-plane kinematic assessment during running, particularly in settings where marker-based systems are less practical. However, the available evidence does not establish MMC as interchangeable with marker-based systems, and further validation across different running conditions, system configurations, and real-world settings is warranted.

## Data Availability

The original contributions presented in the study are included in the article/Supplementary Material, further inquiries can be directed to the corresponding authors.
