# Supplementary material for: Accuracy of markerless motion capture in assessing sagittal-plane lower limb kinematics during running: a systematic review and meta-analysis
Source: Front Bioeng Biotechnol. 2026 Oct 12;14:1915559. doi: 10.3389/fbioe.2026.1915559 (PMC13696929; doi:10.3389/fbioe.2026.1915559)
Supplement: Supplementary file 1 [file Supplementaryfile1.docx]

**Table S1** Database-specific search strategies

| **Database** | **Search Strategy** |
| --- | --- |
| **MEDLINE** | 1.("markerless motion capture"[Title/Abstract] OR "markerless motion analysis"[Title/Abstract] OR "markerless motion tracking"[Title/Abstract] OR "markerless mocap"[Title/Abstract])  2.("marker-based motion capture"[Title/Abstract] OR "marker based motion capture"[Title/Abstract] OR "marker-based motion analysis"[Title/Abstract] OR "marker-based mocap"[Title/Abstract])  3.("running"[Title/Abstract] OR "gait"[Title/Abstract] OR "treadmill"[Title/Abstract])  4.("lower limb"[Title/Abstract] OR "lower extremity"[Title/Abstract] OR "hip"[Title/Abstract] OR "knee"[Title/Abstract] OR "ankle"[Title/Abstract])  5.("sagittal plane"[Title/Abstract] OR "sagittal-plane kinematics"[Title/Abstract])  6.("accuracy"[Title/Abstract] OR "reliability"[Title/Abstract] OR "measurement error"[Title/Abstract] OR "RMSE"[Title/Abstract] OR "RMSD"[Title/Abstract])  7.#1 AND #2 AND #3 AND #4 AND #5 AND #6 |
| **Embase** | 1.('markerless motion capture':ti,ab,kw OR 'markerless motion analysis':ti,ab,kw OR 'markerless motion tracking':ti,ab,kw OR 'markerless mocap':ti,ab,kw)  2.('marker based motion capture':ti,ab,kw OR 'marker-based motion capture':ti,ab,kw OR 'marker based motion analysis':ti,ab,kw OR 'marker-based mocap':ti,ab,kw)  3.('running':ti,ab,kw OR 'gait':ti,ab,kw OR 'treadmill':ti,ab,kw)  4.('lower limb':ti,ab,kw OR 'lower extremity':ti,ab,kw OR 'hip':ti,ab,kw OR 'knee':ti,ab,kw OR 'ankle':ti,ab,kw)  5.('sagittal plane':ti,ab,kw OR 'sagittal plane kinematics':ti,ab,kw)  6.('accuracy':ti,ab,kw OR 'reliability':ti,ab,kw OR 'measurement error':ti,ab,kw OR 'RMSE':ti,ab,kw OR 'RMSD':ti,ab,kw)  7.#1 AND #2 AND #3 AND #4 AND #5 AND #6 |
| **Cochrane Library** | 1.("markerless motion capture" OR "markerless motion analysis" OR "markerless motion tracking" OR "markerless mocap")  2.("marker-based motion capture" OR "marker based motion capture" OR "marker-based motion analysis" OR "marker-based mocap")  3.("running" OR "gait" OR "treadmill")  4.("lower limb" OR "lower extremity" OR "hip" OR "knee" OR "ankle")  5.("sagittal plane" OR "sagittal-plane kinematics")  6.("accuracy" OR "reliability" OR "measurement error" OR "RMSE" OR "RMSD")  7. #1 AND #2 AND #3 AND #4 AND #5 AND #6 |
| **Web of Science** | 1.TS=("markerless motion capture" OR "markerless motion analysis" OR "markerless motion tracking" OR "markerless mocap")  2.TS=("marker based motion capture" OR "marker-based motion capture" OR "marker based motion analysis" OR "marker-based mocap")  3.TS=("running" OR "gait" OR "treadmill")  4.TS=("lower limb" OR "lower extremity" OR "hip" OR "knee" OR "ankle")  5.TS=("sagittal plane" OR "sagittal plane kinematics")  6.TS=("accuracy" OR "reliability" OR "measurement error" OR "RMSE" OR "RMSD")  7.#1 AND #2 AND #3 AND #4 AND #5 AND #6 |
| **Scopus** | 1.TITLE-ABS-KEY("markerless motion capture" OR "markerless motion analysis" OR "markerless motion tracking" OR "markerless mocap")  2.TITLE-ABS-KEY("marker based motion capture" OR "marker-based motion capture" OR "marker based motion analysis" OR "marker-based mocap")  3.TITLE-ABS-KEY("running" OR "gait" OR "treadmill")  4.TITLE-ABS-KEY("lower limb" OR "lower extremity" OR "hip" OR "knee" OR "ankle")  5.TITLE-ABS-KEY("sagittal plane" OR "sagittal plane kinematics")  6.TITLE-ABS-KEY("accuracy" OR "reliability" OR "measurement error" OR "RMSE" OR "RMSD")  7.#1 AND #2 AND #3 AND #4 AND #5 AND #6 |

**Table S2** List of excluded studies

| **Year** | **Title** | **DOI** | **Reason for exclusion** |
| --- | --- | --- | --- |
| 2025 | Marker based and markerless motion capture for equestrian rider kinematic analysis: A comparative study | https://doi.org/10.1016/j.jbiomech.2025.112728 | Not running task |
| 2019 | The development and evaluation of a fully automated markerless motion capture workflow | https://doi.org/10.1001/jama.2019.0922 | Not running task |
| 2024 | AI-smartphone markerless motion capturing of hip, knee, and ankle joint kinematics during countermovement jumps | https://doi.org/10.1002/ejsc.12186 | Not running task |
| 2022 | Development of Smartphone Application for Markerless Three-Dimensional Motion Capture Based on Deep Learning Model | https://doi.org/10.3390/s22145282 | Not running task |
| 2026 | Multi-Patient Vision Transformer for Markerless Tumor Motion Forecasting | https://doi.org/10.3390/biomedicines14030496 | Not running task |
| 2022 | Accuracy of a markerless motion capture system for postural ergonomic risk assessment in occupational practice | https://doi.org/10.1080/10803548.2021.1954791 | Not running task |
| 2024 | Using a Markerless Motion Capture System to Identify Preinjury Differences in Functional Assessments | https://doi.org/10.1055/s-0043-1772238 | Not running task |
| 2025 | Dual-Camera Markerless Motion Capture System for Precise Lower-Limb Kinematic Analysis in Osteoarthritis | https://doi.org/10.1007/s10439-025-03859-z | Not running task |
| 2022 | Accuracy of a markerless motion capture system in estimating upper extremity kinematics during boxing | https://doi.org/10.3389/fspor.2022.939980 | Not running task |
| 2022 | Clothing condition does not affect meaningful clinical interpretation in markerless motion capture | https://doi.org/10.1016/j.jbiomech.2022.111182 | Not running task |
| 2024 | Inter-trial variability is higher in 3D markerless compared to marker-based motion capture: Implications for data post-processing and analysis | https://doi.org/10.1016/j.jbiomech.2024.112049 | Not running task |
| 2023 | Comparison of markerless and marker-based motion capture systems using 95% functional limits of agreement in a linear mixed-effects modelling framework | https://doi.org/10.1038/s41598-023-49360-2 | Not running task |
| 2025 | Clinical integration of markerless motion capture: A multicentre study of gait in knee osteoarthritis | https://doi.org/10.1016/j.jbiomech.2025.112952 | Not running task |
| 2023 | A markerless beam's eye view motion monitoring algorithm based on structure conversion and demons registration in medical image with partial data | https://doi.org/10.1002/mp.16250 | Not running task |
| 2023 | High-resolution multi-shot diffusion-weighted MRI combining markerless prospective motion correction and locally low-rank constrained reconstruction | https://doi.org/10.1002/mrm.29468 | Not running task |
| 2023 | Clinical utility of markerless motion capture for kinematic evaluation of sit-to-stand during 30 s-CST at one year post total knee arthroplasty: a retrospective study | https://doi.org/10.1186/s12891-023-06364-3 | Not running task |
| 2023 | A comparison of three-dimensional kinematics between markerless and marker-based motion capture in overground gait | https://doi.org/10.1016/j.jbiomech.2023.111793 | Not running task |
| 2025 | The Validity and Usability of Markerless Motion Capture and Inertial Measurement Units for Quantifying Dynamic Movements | https://doi.org/10.1249/MSS.0000000000003579 | Not running task |
| 2023 | Validity of artificial intelligence-based markerless motion capture system for clinical gait analysis: Spatiotemporal results in healthy adults and adults with Parkinson's disease | https://doi.org/10.1016/j.jbiomech.2023.111645 | Not running task |
| 2024 | Comparison of kinematics and joint moments calculations for lower limbs during gait using markerless and marker-based motion capture | https://doi.org/10.3389/fbioe.2024.1280363 | Not running task |
| 2023 | Principal component analysis of whole-body kinematics using markerless motion capture during static balance tasks | https://doi.org/10.1016/j.jbiomech.2023.111556 | Not running task |
| 2024 | Objective Falls Risk Assessment Using Markerless Motion Capture and Representational Machine Learning | https://doi.org/10.3390/s24144593 | Not running task |
| 2021 | Artificial Intelligence-Assisted motion capture for medical applications: a comparative study between markerless and passive marker motion capture | https://doi.org/10.1080/10255842.2020.1856372 | Not running task |
| 2023 | Quantitative Comparison of Hand Kinematics Measured with a Markerless Commercial Head-Mounted Display and a Marker-Based Motion Capture System in Stroke Survivors | https://doi.org/10.3390/s23187906 | Not running task |
| 2024 | Assessment of Hip and Lumbar Spine Range of Motion After Total Hip Arthroplasty Using a Single Camera Markerless System | https://doi.org/10.7759/cureus.65875 | Not running task |
| 2023 | Reliability and Concurrent Validity of a Markerless, Single Camera, Portable 3D Motion Capture System for Assessment of Glenohumeral Mobility | https://doi.org/10.26603/001c.88003 | Not running task |
| 2026 | Reliability assessment of markerless technologies in biomechanical motion analysis: a performance comparison | https://doi.org/10.3389/fspor.2025.1712332 | Not running task |
| 2022 | Repeatability of Motion Health Screening Scores Acquired from a Three-Dimensional Markerless Motion Capture System | https://doi.org/10.3390/jfmk7030065 | Not running task |
| 2022 | Absolute Reliability of Gait Parameters Acquired With Markerless Motion Capture in Living Domains | https://doi.org/10.3389/fnhum.2022.867474 | Not running task |
| 2025 | From Wearable Sensor Networks to Markerless Motion Capture for Instrumental-Based Biomechanical Risk Assessment in Lifting Activities | https://doi.org/10.3390/s25247427 | Not running task |
| 2025 | Validity of AI-Driven Markerless Motion Capture for Spatiotemporal Gait Analysis in Stroke Survivors | https://doi.org/10.3390/s25175315 | Not running task |
| 2024 | Exploring stroke survivors' and physiotherapists' perspectives of the potential for markerless motion capture technology in community rehabilitation | https://doi.org/10.1186/s12984-024-01467-x | Not running task |
| 2025 | Smartphone-Based Markerless Motion Capture for Accessible Rehabilitation: A Computer Vision Study | https://doi.org/10.3390/s25175428 | Not running task |
| 2025 | Concurrent validity of Theia3D markerless motion capture for detecting sagittal kinematic differences between gait speeds | https://doi.org/10.1080/02640414.2025.2513151 | Not running task |
| 2025 | Optimisation and Comparison of Markerless and Marker-Based Motion Capture Methods for Hand and Finger Movement Analysis | https://doi.org/10.3390/s25041079 | Not running task |
| 2022 | Towards Single Camera Human 3D-Kinematics | https://doi.org/10.3390/s23010341 | No hip, knee, or ankle RMSE not available |
| 2021 | Wearable Motion Capture System Evaluation for Biomechanical Studies for Hip Joints | https://doi.org/10.1115/1.4049199 | No hip, knee, or ankle RMSE not available |
| 2023 | Predictive utility of commercial grade technologies for assessing musculoskeletal injury risk in US Marine Corps Officer candidates | https://doi.org/10.3389/fphys.2023.1088813 | No hip, knee, or ankle RMSE not available |
| 2025 | Relationship between volleyball spike jump height and lower limb kinetics is stronger for orientation leg than rear leg | https://doi.org/10.1080/14763141.2025.2458491 | No hip, knee, or ankle RMSE not available |
| 2025 | Automatic infant 2D pose estimation from videos: Comparing seven deep neural network methods | https://doi.org/10.3758/s13428-025-02816-x | No hip, knee, or ankle RMSE not available |
| 2021 | Artificial Intelligence-Assisted motion capture for medical applications: a comparative study between markerless and passive marker motion capture | https://doi.org/10.1080/10255842.2020.1856372 | No hip, knee, or ankle RMSE not available |
| 2025 | Comparison of kinematics and kinetics between OpenCap and a marker-based motion capture system in cycling | https://doi.org/10.1016/j.compbiomed.2025.110295 | No hip, knee, or ankle RMSE not available |
| 2025 | Evaluation of the Ergomechanic Markerless Motion Capture System for Lower Body Kinematics During Standing, Squatting, and Walking | https://doi.org/10.1115/1.4069821 | No hip, knee, or ankle RMSE not available |
| 2024 | Evaluation of drop vertical jump kinematics and kinetics using 3D markerless motion capture in a large cohort | https://doi.org/10.3389/fbioe.2024.1426677 | No hip, knee, or ankle RMSE not available |
| 2024 | Using a Markerless Motion Capture System to Identify Preinjury Differences in Functional Assessments | https://doi.org/10.1055/s-0043-1772238 | No hip, knee, or ankle RMSE not available |
| 2026 | Smartphone-Based Markerless Motion Capture for Spatiotemporal Gait Assessment: Applied Within-Session Reliability and Comparability of OpenCap Versus OptoGait | https://doi.org/10.3390/s26041234 | No hip, knee, or ankle RMSE not available |
| 2025 | Anatomical-Marker-Driven 3D Markerless Human Motion Capture | https://doi.org/10.1109/JBHI.2024.3424869 | Incompatible motion capture system |
| 2019 | Optimal marker set assessment for motion capture of 3D mimic facial movements | https://doi.org/10.1016/j.jbiomech.2019.06.012 | Incompatible motion capture system |
| 2023 | Efficacy of Marker-Based Motion Capture for Respiratory Cycle Measurement: A Comparison with Spirometry | https://doi.org/10.3390/s23249736 | Incompatible motion capture system |
| 2018 | Generic Content-Based Retrieval of Marker-Based Motion Capture Data | https://doi.org/10.1109/TVCG.2017.2702620 | Incompatible motion capture system |
| 2020 | Automated gap-filling for marker-based biomechanical motion capture data | https://doi.org/10.1080/10255842.2020.1789971 | Incompatible motion capture system |
| 2024 | Deep-Learning-Based Recovery of Missing Optical Marker Trajectories in 3D Motion Capture Systems | https://doi.org/10.3390/bioengineering11060560 | Incompatible motion capture system |
| 2026 | Uncertainty estimation in marker-based motion capture of the tennis serve | https://doi.org/10.1016/j.jbiomech.2025.113119 | Incompatible motion capture system |
| 2025 | Development of Marker-Based Motion Capture Using RGB Cameras: A Neural Network Approach for Spherical Marker Detection | https://doi.org/10.3390/s25175228 | Incompatible motion capture system |
| 2021 | Comparison of the Performance of the Leap Motion Controller^TM^ with a Standard Marker-Based Motion Capture System | https://doi.org/10.3390/s21051750 | Incompatible motion capture system |
| 2022 | Applications and limitations of current markerless motion capture methods for clinical gait biomechanics | https://doi.org/10.7717/peerj.12995 | Review or non-original study |
| 2025 | Markerless Motion Analysis Using New Digital Technology | https://doi.org/10.1298/ptr.R0037 | Review or non-original study |
| 2023 | Technological advancements in the analysis of human motion and posture management through digital devices | https://doi.org/10.5312/wjo.v12.i7.467 | Review or non-original study |
| 2024 | Accuracy, Validity, and Reliability of Markerless Camera-Based 3D Motion Capture Systems versus Marker-Based 3D Motion Capture Systems in Gait Analysis: A Systematic Review and Meta-Analysis | https://doi.org/10.3390/s24113686 | Review or non-original study |
| 2021 | Applications of Pose Estimation in Human Health and Performance across the Lifespan | https://doi.org/10.3390/s21217315 | Review or non-original study |
| 2022 | Walking as a Window to the Brain: Redefining Gait in Neurology | https://doi.org/10.3390/medsci14030338 | Review or non-original study |
| 2025 | Challenges in Combining EMG, Joint Moments, and GRF from Marker-Less Video-Based Motion Capture Systems | https://doi.org/10.3390/bioengineering12050461 | Review or non-original study |
| 2025 | AI Video Analysis in Parkinson's Disease: A Systematic Review of the Most Accurate Computer Vision Tools for Diagnosis, Symptom Monitoring, and Therapy Management | https://doi.org/10.3390/s25206373 | Review or non-original study |
| 2022 | A multimodal Parkinson quantification by fusing eye and gait motion patterns, using covariance descriptors, from non-invasive computer vision | https://doi.org/10.1016/j.cmpb.2021.106607 | Review or non-original study |
| 2019 | Methodological considerations for kinematic analysis of upper limbs in healthy and poststroke adults Part II: a systematic review of motion capture systems and kinematic metrics | https://doi.org/10.1080/10749357.2019.1611221 | Review or non-original study |
| 2024 | The applicability of markerless motion capture for clinical gait analysis in children with cerebral palsy | https://doi.org/10.1038/s41598-024-62119-7 | Incomplete or missing data |
| 2020 | Evaluation of 3D Markerless Motion Capture Accuracy Using OpenPose With Multiple Video Cameras | https://doi.org/10.3389/fspor.2020.00050 | Incomplete or missing data |
| 2024 | Synchronised Video, Motion Capture and Force Plate Dataset for Validating Markerless Human Movement Analysis | https://doi.org/10.1038/s41597-024-04077-3 | Incomplete or missing data |
| 2021 | Validation of a Commercially Available Markerless Motion-Capture System for Trunk and Lower Extremity Kinematics During a Jump-Landing Assessment | https://doi.org/10.4085/1062-6050-0023.20 | Incomplete or missing data |

**Figure S1** Subgroup analysis of measurement error for MMC systems with a sampling frame rate of ≤60 Hz.

**Figure S2** Subgroup analysis of measurement error for MMC systems with a sampling frame rate of >60 Hz.

**Figure S3.** Subgroup analysis of measurement error according to the number of cameras, with ≤8 cameras.

**Figure S4.** Subgroup analysis of measurement error according to the number of cameras, with >8 cameras.

**Figure S5.** Subgroup analysis of measurement error during overground running.

**Figure S6.** Subgroup analysis of measurement error during treadmill running.

**Figure S7.** Forest plots of sensitivity analyses for sagittal-plane RMSE of the hip, knee, and ankle joints.
